# Genetic prediction of ICU hospitalization and mortality in COVID‐19 patients using artificial neural networks

**DOI:** 10.1111/jcmm.17098

**Published:** 2022-01-22

**Authors:** Panagiotis G. Asteris, Eleni Gavriilaki, Tasoula Touloumenidou, Evaggelia‐Evdoxia Koravou, Maria Koutra, Penelope Georgia Papayanni, Alexandros Pouleres, Vassiliki Karali, Minas E. Lemonis, Anna Mamou, Athanasia D. Skentou, Apostolia Papalexandri, Christos Varelas, Fani Chatzopoulou, Maria Chatzidimitriou, Dimitrios Chatzidimitriou, Anastasia Veleni, Evdoxia Rapti, Ioannis Kioumis, Evaggelos Kaimakamis, Milly Bitzani, Dimitrios Boumpas, Argyris Tsantes, Damianos Sotiropoulos, Anastasia Papadopoulou, Ioannis G. Kalantzis, Lydia A. Vallianatou, Danial J. Armaghani, Liborio Cavaleri, Amir H. Gandomi, Mohsen Hajihassani, Mahdi Hasanipanah, Mohammadreza Koopialipoor, Paulo B. Lourenço, Pijush Samui, Jian Zhou, Ioanna Sakellari, Serena Valsami, Marianna Politou, Styliani Kokoris, Achilles Anagnostopoulos

**Affiliations:** ^1^ Computational Mechanics Laboratory School of Pedagogical and Technological Education Athens Greece; ^2^ Hematology Department BMT Unit G Papanicolaou Hospital Thessaloniki Greece; ^3^ Biomedical Laboratories Athens Greece; ^4^ Rheumatology and Clinical Immunology Unit University General Hospital ‘Attikon’ Athens Greece; ^5^ Microbiology Department Aristotle University of Thessaloniki Greece; ^6^ Biomedical Sciences Alexander Campus International Hellenic University Thessaloniki Greece; ^7^ Infectious Disease Committee G Papanicolaou Hospital Thessaloniki Greece; ^8^ Laboratory of Hematology and Hospital Blood Transfusion Department University General Hospital ‘Attikon’ NKUA Medical School Athens Greece; ^9^ Respiratory Failure Department G Papanicolaou Hospital Aristotle University of Thessaloniki Thessaloniki Greece; ^10^ 1^st^ Intensive Care Unit G Papanicolaou Hospital Thessaloniki Greece; ^11^ Gastroenterology Department Hellenic Red Cross Hospital Athens Greece; ^12^ Cath and EP Laboratory/Covid Intensive Care Unit Hellenic Red Cross Hospital Athens Greece; ^13^ Department of Civil Engineering Faculty of Engineering University of Malaya Kuala Lumpur Malaysia; ^14^ Department of Civil, Environmental, Aerospace and Materials Engineering University of Palermo Palermo Italy; ^15^ Faculty of Engineering & Information Technology University of Technology Sydney Ultimo NSW Australia; ^16^ Department of Mining Engineering Faculty of Engineering Urmia University Urmia Iran; ^17^ Institute of Research and Development Duy Tan University Da Nang Vietnam; ^18^ Faculty of Civil and Environmental Engineering Amirkabir University of Technology Tehran Iran; ^19^ Department of Civil Engineering ISISE University of Minho Guimarães Portugal; ^20^ Department of Civil Engineering National Institute of Technology Patna Patna Bihar India; ^21^ School of Resources and Safety Engineering Central South University Changsha China; ^22^ Hematology Laboratory ‐ Blood Bank Aretaieion Hospital School of Medicine NKUA Athens Greece

**Keywords:** artificial intelligence, complement, complement inhibition, COVID‐19, genetic susceptibility, SARS‐CoV2

## Abstract

There is an unmet need of models for early prediction of morbidity and mortality of Coronavirus disease‐19 (COVID‐19). We aimed to a) identify complement‐related genetic variants associated with the clinical outcomes of ICU hospitalization and death, b) develop an artificial neural network (ANN) predicting these outcomes and c) validate whether complement‐related variants are associated with an impaired complement phenotype. We prospectively recruited consecutive adult patients of Caucasian origin, hospitalized due to COVID‐19. Through targeted next‐generation sequencing, we identified variants in complement factor H/*CFH*, *CFB*, *CFH*‐*related*, *CFD*, *CD55*, *C3*, *C5*, *CFI*, *CD46*, *thrombomodulin*/*THBD*, and *A Disintegrin and Metalloproteinase with Thrombospondin motifs (ADAMTS13)*. Among 381 variants in 133 patients, we identified 5 critical variants associated with severe COVID‐19: rs2547438 (*C3*), rs2250656 (*C3*), rs1042580 (*THBD*), rs800292 (*CFH*) and rs414628 (*CFHR1*). Using age, gender and presence or absence of each variant, we developed an ANN predicting morbidity and mortality in 89.47% of the examined population. Furthermore, THBD and C3a levels were significantly increased in severe COVID‐19 patients and those harbouring relevant variants. Thus, we reveal for the first time an ANN accurately predicting ICU hospitalization and death in COVID‐19 patients, based on genetic variants in complement genes, age and gender. Importantly, we confirm that genetic dysregulation is associated with impaired complement phenotype.

## 1 INTRODUCTION

Coronavirus disease‐19 (COVID‐19) caused by severe acute respiratory syndrome coronavirus‐2 (SARS‐CoV‐2) has led to unprecedented morbidity and mortality worldwide.^1^ Although vaccination against SARS‐CoV‐2 has positively impacted the course of this pandemic,^2^ the unmet need of reducing morbidity and mortality due to severe COVID‐19, especially in special populations, remains. Accumulating evidence suggests that SARS‐CoV‐2 induces a vicious cycle of immune dysfunction, endothelial injury, complement activation and microangiopathy. In particular, severe COVID‐19 is a multisystemic vascular disease characterized by endothelial dysfunction.^3^, ^4^ Therefore, an improved understanding of endothelial dysfunction and complement activation is of utmost importance.

In this context, several groups worldwide have shown evidence of complement activation in experimental and clinical studies of severe COVID‐19.^5^, ^6^, ^7^, ^8^, ^9^, ^10^, ^11^, ^12^, ^13^ Based on the paradigm of genetic susceptibility in complement‐mediated disorders or complementopathies,^14^ our group and other researchers have suggested genetic susceptibility identifying complement genetic variants in COVID‐19 patients.^15^, ^16^, ^17^ In parallel, complement inhibitors have been safe and effective in severe COVID‐19 during the first wave.^18^ Encouraging results have been reported in case series for terminal complement inhibition with eculizumab,^19^ C3 inhibition with the AMY‐101,^20^ C1 inhibition with conestat alpha^21^ and lectin pathway inhibition with narsoplimab.^22^ The comparison of AMY‐101 to eculizumab suggested a broader involvement of C3 in thromboinflammation.^23^ Based on promising data, randomized controlled trials are ongoing for AMY‐191 and eculizumab in severe COVID‐19 (NCT04346797 and NCT04395456). Interim analysis is still pending on the paused phase 3 study of the long‐acting terminal complement inhibitor ravulizumab. Nevertheless, several issues need to be considered for the wider use of complement inhibitors in COVID‐19, including the complex setting of inflammatory responses in COVID‐19, the cost and the limitation of drug accessibility. All require proper selection of patients that would potentially benefit from complement inhibition.

Taking into account the constantly evolving COVID‐19 landscape due to vaccination and viral mutations, there is an unmet clinical need of a prediction tool based on robust variables. Therefore, we aimed initially to identify critical complement‐related genetic variants predicting severe COVID‐19. Then, for the first time in COVID‐19 patients, we sought to develop an artificial neural network (ANN) that predicts morbidity and mortality incorporating genetic variants and defining characteristics such as age and gender, but also to validate that the identified complement‐related variants are associated with an impaired complement phenotype who might benefit from complement inhibitors.

## 2 METHODS

### 2.1 Artificial neural networks

Development of artificial neural networks (ANNs) is based on the concept of the biological neural network of the human brain and was initially used for medicine research purposes to simulate strongly non‐linear relationships between numerous input and output parameters.^24^, ^25^, ^26^, ^27^, ^28^, ^29^, ^30^ ANN models were subsequently introduced into the wider context of engineering disciplines,^31^, ^32^ which significantly enriched the mathematical background underpinning ANN. At present, ANNs are widely employed on an interdisciplinary interface between various disciplines.^17^ A few studies employing ANNs have also emerged focussing on COVID‐19 prediction problems.^33^, ^34^, ^35^, ^36^, ^37^, ^38^, ^39^, ^40^

**FIGURE 1:**
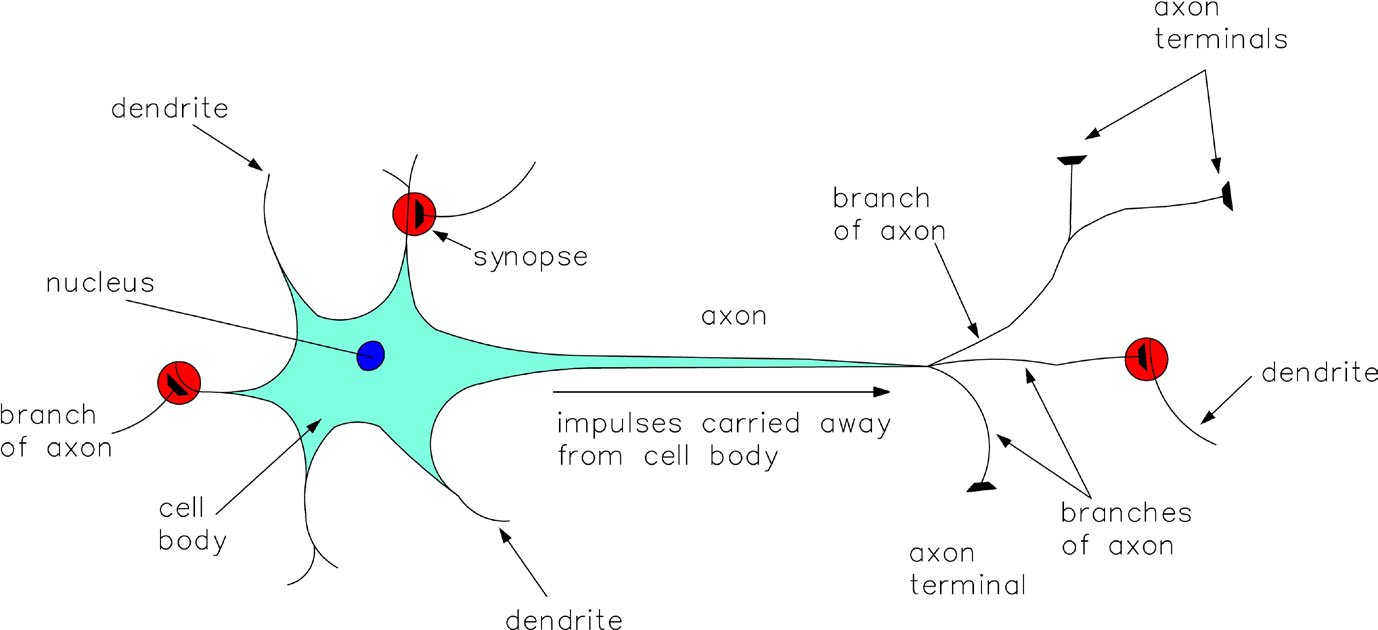
Typical biological neuron structure. The artificial neural network mathematical models have an analogous simplified architecture resembling the structure of neurons in the biological prototype

The basic building block of ANNs is the artificial neuron, which is a mathematical model mimicking the behaviour of the biological neuron (Figure 1). Information is passed onto the artificial neuron as an input parameter and is then processed using a mathematical function to derive an output which determines the behaviour of the neuron (similar to the fire‐or‐not situation of the biological neuron). Before the information enters the neuron, it is weighted in order to simulate the random nature of the biological neuron. A group of such neurons comprise an ANN, similar to the structure of biological neural networks. To define an ANN, (i) the architecture of the ANN, (ii) the training algorithm used during the ANN’s training stage and (iii) the mathematical functions underpinning the mathematical model are required.

The architecture of the ANN defines how the artificial neurons are organized and how the information flows within the network. For example, if the neurons are organized in more than one layer, then the network is called a multilayer ANN. The training stage can be considered as a function minimization problem, in which the optimum weight values need to be determined by minimizing an error function.

### 2.2 Patient population

We prospectively recruited consecutive adult patients of Caucasian origin, hospitalized due to COVID‐19 in our referral centres (G Papanicolaou, Attikon Hospital) (April‐December 2020). RT‐PCR (reverse‐transcriptase polymerase chain reaction) confirmed SARS‐COV2 infection. Patients’ history and course were documented by treating physicians following patients up to discharge or death. Our study was approved by Institutional Review Boards (IRBs) of referral centres and conducted according to the Declaration of Helsinki.

**FIGURE 2:**
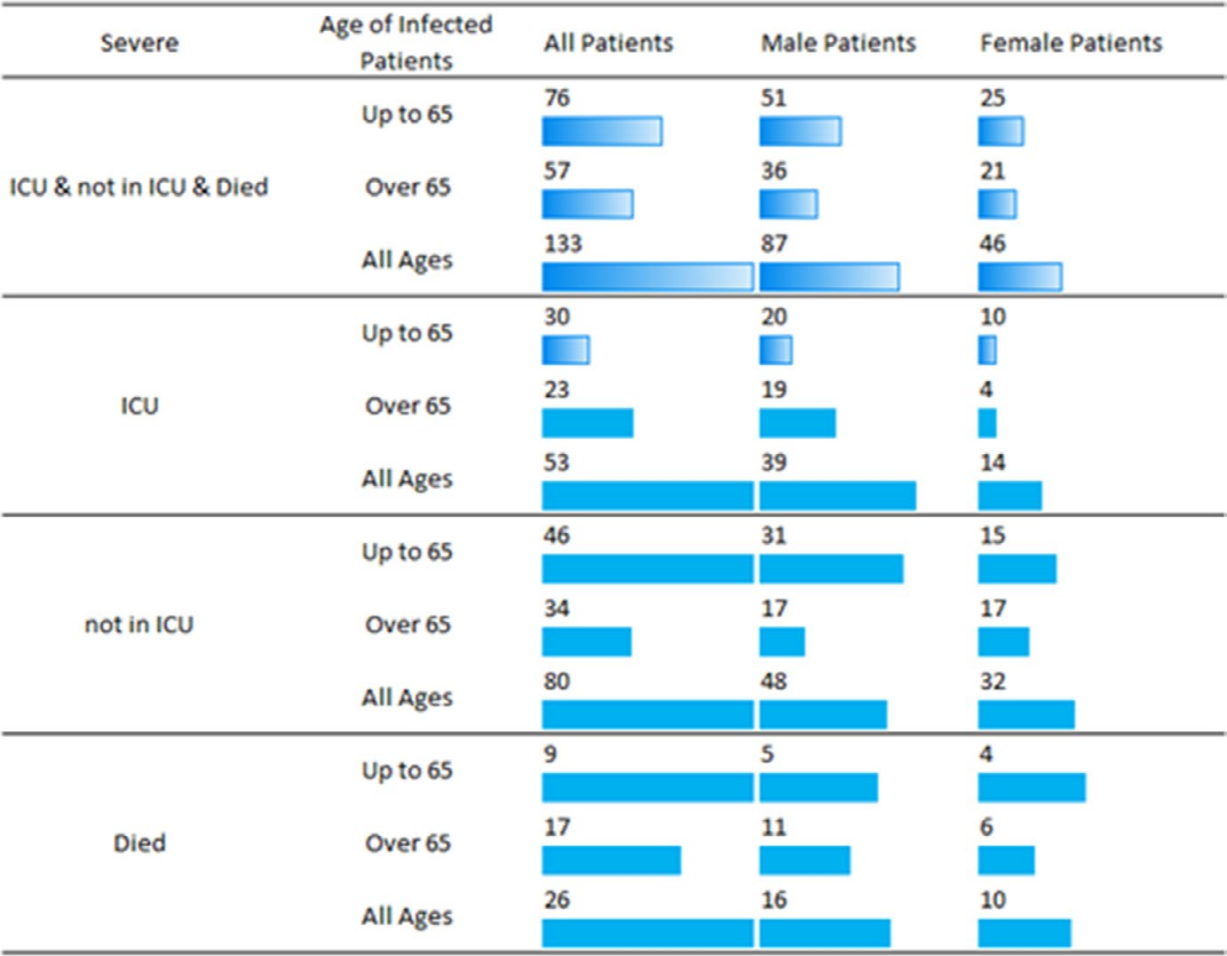
Study population characteristics categorized by age, gender and infection severity (requiring or not requiring intensive care unit (ICU), mortality)

We studied 133 COVID‐19 patients, 80 hospitalized in COVID‐19 general ward (GW‐not in ICU) and 53 hospitalized in intensive care units (ICU). Figure 2 presents patients characteristics according to age, gender and disease severity.

### 2.3 Genetic studies

Genetic studies were performed as previously described.^17^ Briefly, peripheral blood samples were used to isolate genomic DNA that underwent next‐generation sequencing (NGS, MiniSeq, Illumina, San Diego, California) analysis with our customized gene panel for complement factor H/*CFH*, *CFB*, *CFH*‐*related*, *CFD*, *CD55*, *C3*, *C5*, *CFI*, *CD46*, *thrombomodulin*/*THBD*, and *A Disintegrin and Metalloproteinase with Thrombospondin motifs (ADAMTS13)*. DesignStudio (Illumina, San Diego, California) was used for probes design, in order to cover all exonic regions spanning 15 bases into introns (coverage 98%).

### 2.4 Functional assessment of complement activation

Plasma was isolated from EDTA tubes collected at hospitalization for non‐ICU patients or at ICU admission for ICU patients and stored immediately at −80^0^C. Taking into account the difficulties of functional complement assessment in clinical laboratories,^41^ we studied markers that would be potentially accessible in everyday clinical practice or the setting of a clinical trial. Therefore, we measured THBD (R&D, Bio‐Techne), C3a (Invitrogen, Thermo Fisher Scientific) and C5a (Affymetrix, Bender Medsystems) using commercially available ELISA assays. Statistical comparison of continuous variables between groups was performed using the statistical program SPSS 23.0 (IBM SPSS Statistics for Windows, Version 23.0.; IBM Corp). Descriptive statistics were described using median (interquartile range) or mean ±standard deviation for continuous variables according to normality, and frequency for categorical variables. Continuous variables were compared using t test or Mann‐Whitney, according to normality.

### 2.5 Criteria for the optimum database filtering

Given the essentially infinite number of available variant combinations (in excess of yotta = 10^24^), advanced computational analysis tools were required to correlate the effect of variant combinations with the severity of COVID‐19 infection. To reduce the computational complexity associated with the significant number of available variants, a pre‐defined set of criteria was used to identify the minimum number of required variants, which are mainly associated with severe COVID‐19 infection.

**Criterion I:**Variants which are present in more than 90% or less than 10% of COVID‐19 infected patients are considered as non‐associated with COVID‐19 severity.
**Criterion II:**From the remaining available variant combinations, single variant types associated with severe complications of COVID‐19 infection are investigated. This is achieved using the following II.I and II.II Criteria.
**Criterion II.Ι:**Variants are identified, which are present in the 90% of COVID‐19 patients requiring intensive care unit (ICU) and at the same time are not present in the 90% of patients not requiring ICU hospitalization.
**Criterion II.ΙI:**Variants are identified, which are not present in the 90% of COVID‐19 patients requiring ICU and at the same time are present in the 90% of COVID‐19 patients not requiring ICU. Criterion II.II is essentially the reverse of Criterion II.I.

This constraint was driven by the computational requirements during the training and development of ANN models. In this study, different architectures of ANNs were trained and developed and the main stages are summarized in the results section, later in the text.

## 3 RESULTS

### 3.1 Genetic analysis

We identified a total of 381 variants, ranging from 40 to 101 per patient (mean value 71 and standard deviation 12). The database of variants is presented as supplementary material in the supplemental excel file entitled variants Database of 133 COVID‐19 Patients.

**FIGURE 3:**
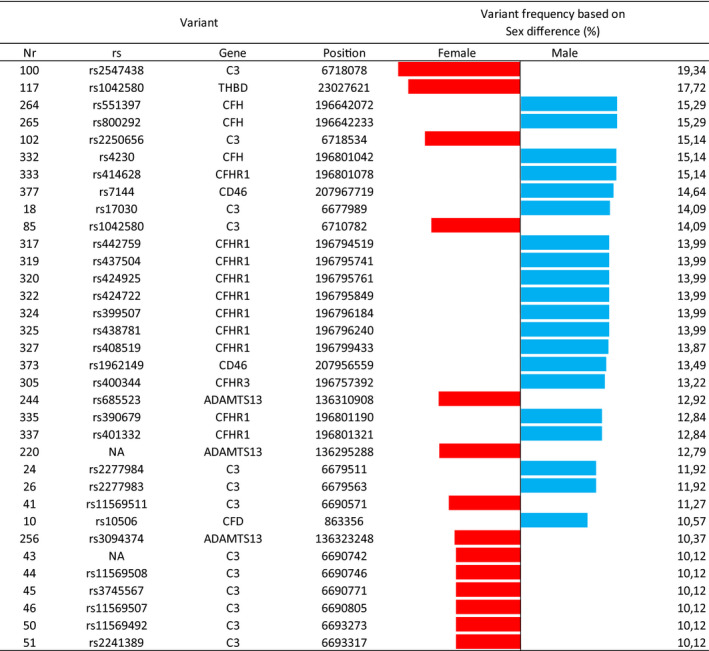
Variant frequency distribution based on gender. The variants satisfying Criterion II are 15% more present in male than in female patients and the reverse (red and blue bars denote higher percentage variant presence in female and male patients respectively)

Using the pre‐defined set of criteria, a total of 381 variants are reduced to 5 critical variants, which are mainly associated with severe COVID‐19 infection (morbidity and mortality), as shown in Table 1: rs2547438 (*C3*), rs2250656 (*C3*), rs1042580 (*THBD*), rs800292 (*CFH*) and rs414628 (*CFHR1*). Variant characteristics are shown in detail in Supplementary Table 1. Interestingly, Figure 3 shows that variants satisfying Criterion II are by 15% more present in male than in female patients and the reverse.

**TABLE 1:** Prediction of COVID‐19 infection severity using the 5 critical variants obtained from criteria I and II

Ranking	Rs	Gene	Position	Percentage prediction									Criterion
				In ICU (53)			Not in ICU (80)			In ICU and not in ICU (133)			
				All	Male	Female	All	Male	Female	All	Male	Female	
(1)	(2)	(3)	(4)	(5)	(6)	(7)	(8)	(9)	(10)	(11)	(12)	(13)	(14)
5	rs2547438	C3	6718078	75.47	79.49	64.29	35.00	27.08	46.88	51.13	50.57	52.17	II
2	rs2250656	C3	6718534	62.26	71.79	35.71	52.50	52.08	53.13	56.39	60.92	47.83	II
1	rs1042580	THBD	23027621	49.06	56.41	28.57	66.25	62.50	71.88	59.40	59.77	58.70	II
4	rs800292	CFH	196642233	37.74	38.46	35.71	65.00	56.25	78.13	54.14	48.28	65.22	I
3	rs414628	CFHR1	196801078	62.26	64.10	57.14	52.50	45.83	62.50	56.39	54.02	60.87	I

Note

Criterion I: If variant (0) is present, then no ICU is required, whereas if variant (1) is not present, then ICU is required.

Criterion II: If variant (0) is present, then ICU is required, whereas if variant (1) is not present, then ICU is not required.

THBD: thrombomodulin; CFH: complement factor H; CFHR: complement factor H‐related; ICU: intensive care unit.

The number of variant combinations is defined as follows:

(1) $$\text{Number of patterns}=\sum_{i=1}^{\mathrm{npv}}\left(\begin{array}{c} \mathrm{nv}\\ \mathrm{npv} \end{array}\right)=\sum_{i=1}^{\mathrm{npv}}\frac{nv!}{i!\left(nv‐i\right)!}$$

where nv is the number of variants and npv is the number of variant patterns.

In this study, nv = 381 number of variants were investigated for a 133 COVID‐19 patient sample.

### 3.2 Development of ANNs

**FIGURE 4:**
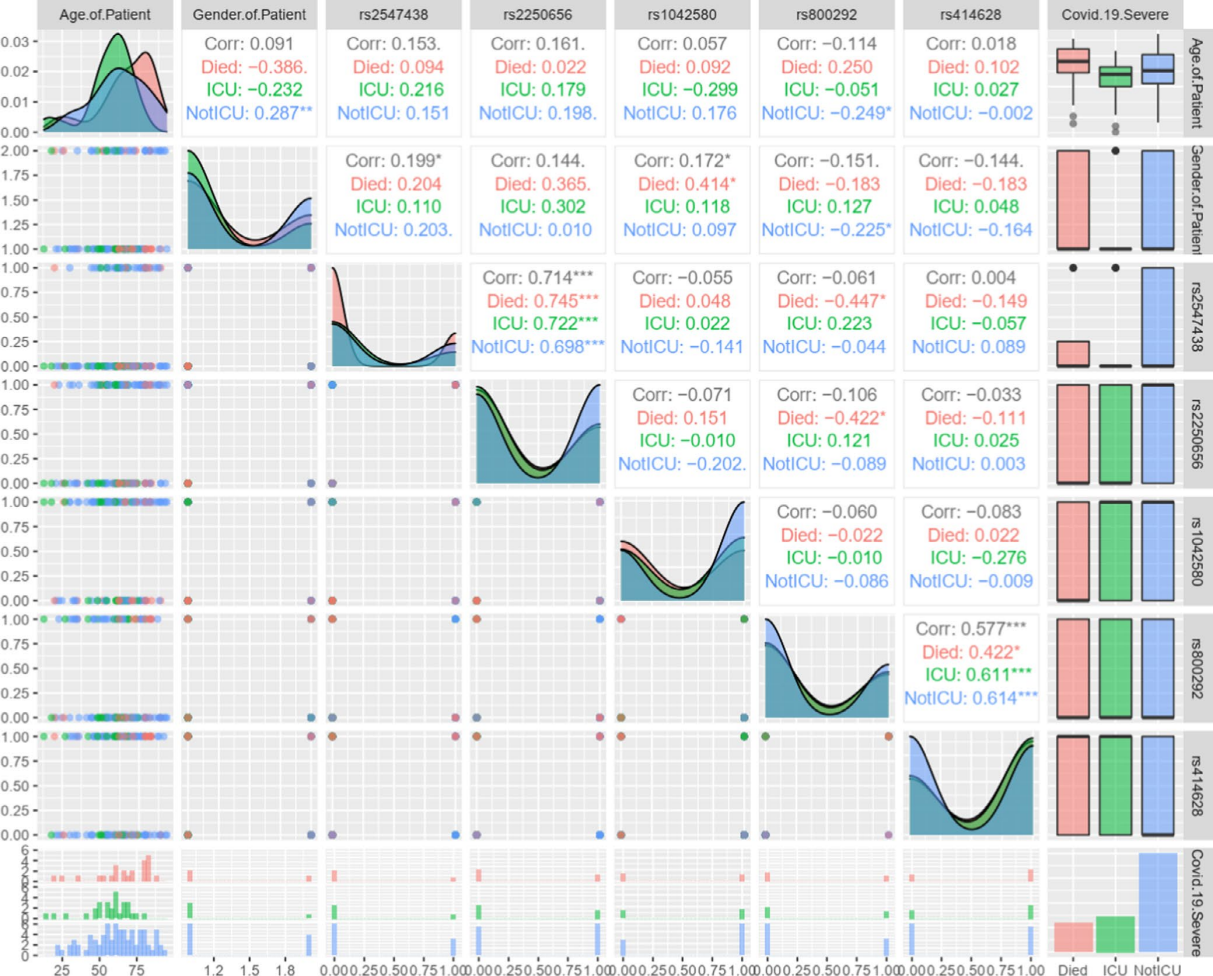
Scatterplot matrix of COVID‐19 samples for the seven input parameters. Each input parameter is plotted against every other one, with different plot types drawn depending on the pair combination. Especially, the diagonal facets show the distributions of values for each parameter, ignoring all others. Along the right column, box plots show the distribution of each continuous parameter against the COVID‐severe cases. The same is depicted in histogram form at the bottom row. Finally, dot plots show the relationships between parameter pairs

The database used in this research comprised of 133 data sets, with each data set containing 7 input parameters (age, gender and 5 parameters indicating the presence or absence of each of the 5 critical variants). Figure 4 illustrates a statistical analysis of the selected input parameters.

The 133 data sets were divided into three separate sets. Specifically, 89 of the 133 (66.92%) data sets were designated as training data sets, 22 of the 133 (16.54%) as validation data sets, while the remaining 22 (16.54%) data sets were used as testing data sets. The assignment of patients to the various data sets was algorithmic and pseudo‐random in nature, in order to prevent any potential bias. It was ensured that the composition of the final data sets in terms of input parameters was representative of the entire population.

For the training and the development of ANNs, it is necessary to define a number of parameters, related to their architecture and algorithmic implementation. Rather than relying on expertise or trial‐and‐error approaches, a number of alternatives were examined for these parameters, leading to the training and development of a plethora of possible ANN model implementations. Supplementary Table 2 presents all the parameters considered for the development of multiple alternative ANN architectures in the present study. ANNs with one or two hidden layers were developed and trained. The number of neurons per hidden layer ranged from 1 to 30, with an incremental step of 1. Ten different initial values of weights and biases were applied for each architecture. Two functions, the mean square error (MSE) and sum square error (SSE) functions, were used as cost functions, during the training and validation process. Ten functions were used as transfer or activation functions. Considering that the implementation of 10 different transfer function results in 1000 different ANNs, different dual combinations of the 10 transfer functions were investigated. During the training stage of ANNs, the 133 data sets were used with and without normalization techniques. When normalization of the data was conducted, the minmax normalization technique in the range [0.10, 0.90] and [−1.00, 1.00] Zscore normalization technique were implemented.

The Levenberg‐Marquardt algorithm was used for the training of the ANNs. Overall, the alternative parameters, examined for the ANN development phase, resulted in the creation and evaluation of 75.000.000 different ANN models, which were ranked according to the prediction accuracy of the COVID‐19 severity.

**FIGURE 5:**
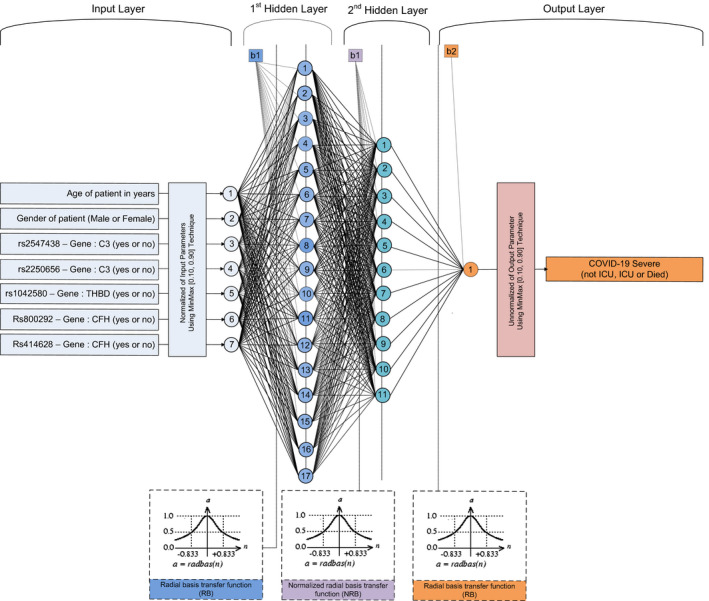
Architecture of the optimum ANN model. The input layer consists of seven parameters (input) which are age and gender of patient, and five crucial variants, while the output layer consists of a single parameter (output) which is the infection severity (requiring or not requiring intensive care unit, fatality). The two hidden layers consist of 17 and 11 neurons respectively. To the bottom of the schematic, the optimum transfer functions for the first hidden layer, second hidden layer and output layer are the radial basis transfer function, normalized radial basis transfer function and radial basis transfer function respectively

**FIGURE 6:**
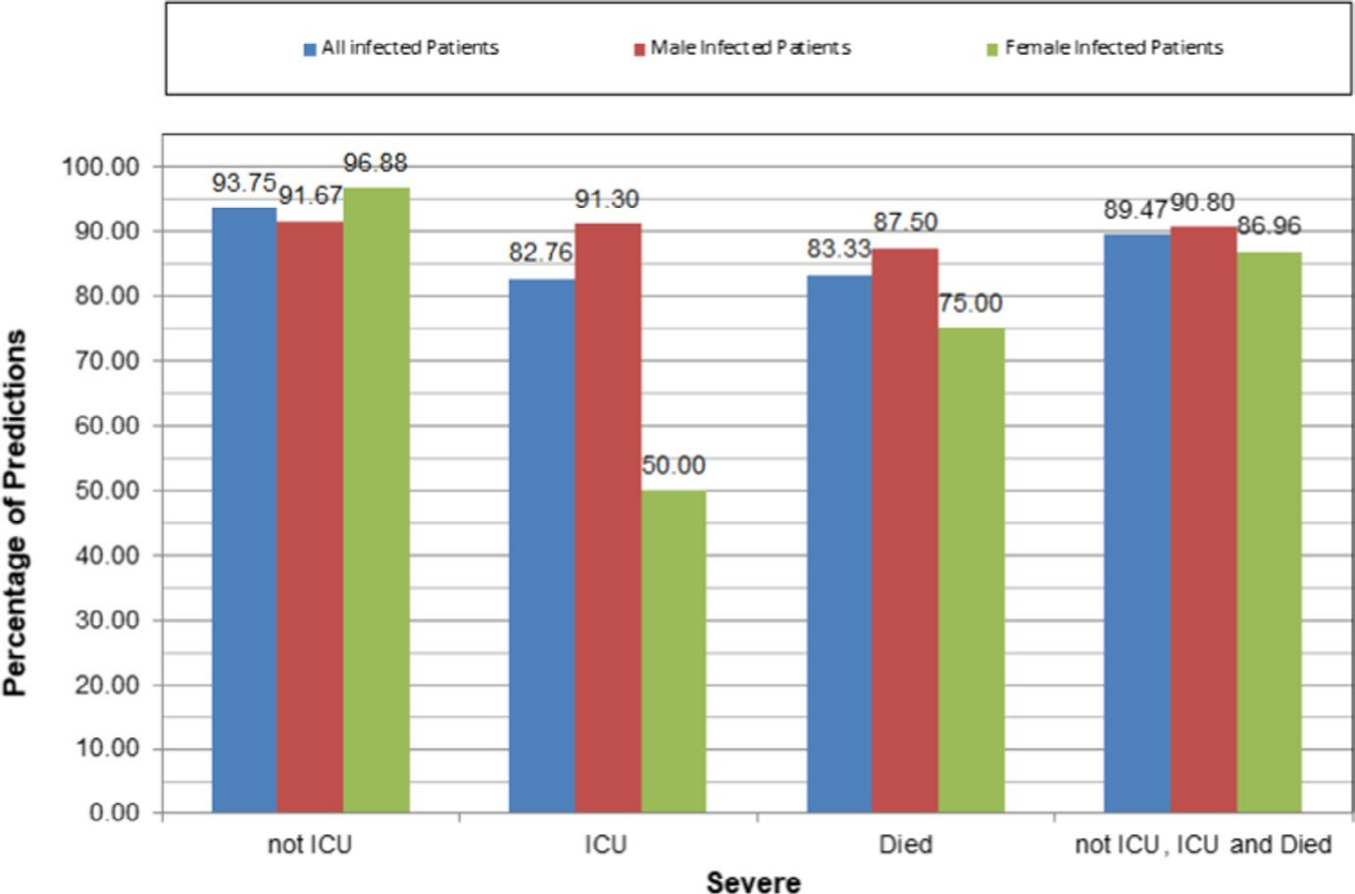
Percentage predictions of COVID‐19 severity based on the optimum ANN model. The first group of three column bars represents the correct predictions of the ANN model for patients that did not require ICU treatment (achieving more than 93% successful predictions). The percentage of successful predictions is better for the female patients (more than 96%), while for male ones the respective percentage is 91.67%. The last group of three column bars represents the correct predictions of the ANN model for all cases of patient infection severity (requiring or not requiring intensive care unit and died). The percentage of successful predictions is more than 90% for male patients and close to 90% for female ones, while the combined percentage is 89.47%. The remaining groups of column bars report the respective results for the patients requiring ICU and those who have died

The architecture of the ANN with the highest prediction accuracy is presented in Figure 5; it consists of 2 hidden layers with 17 neurons in the first hidden layer and 11 neurons in the second hidden layer. The optimum ANN predicts successfully COVID‐19 severity (not ICU, ICU or mortality) in 89.47% of the study population, which corresponds to 90.8% male and 86.96% female patients (Figure 6).

### 3.3 Functional assessment of complement activation

THBD values were significantly increased in patients requiring ICU hospitalization compared to non‐ICU patients (median 2.3, interquartile range [1.6] versus 1.4 [0.79] ng/ml, *p *= 0.025) and patients harbouring the rs1042580 (*THBD*) variant (*p *= 0.032). Similarly, C3a values were significantly increased in patients requiring ICU hospitalization compared to non‐ICU patients (410 [14.1] versus 312 [19.1] ng/ml, *p *= 0.035) and trended towards a significant increase in patients harbouring one of the two critical C3 alleles (rs2547438 or rs2250656, *p *= 0.092). Despite increased levels of C5a in ICU patients, this difference did not reach statistical significance (72.1 [7.2] versus 43.4 [11.3] ng/ml, *p *= 0.244).

## 4 DISCUSSION

We reveal for the first time an ANN able to accurately predict ICU hospitalization and death in COVID‐19 patients, based on genetic variants in complement genes, age and gender. Importantly, we also confirm that genetic dysregulation is associated with an impaired complement phenotype.

Complement‐related genetic variants have been investigated in limited COVID‐19 studies.^15^, ^16^, ^17^ Valenti et al. have recently identified a variant (rs11385942) predisposing to severe COVID‐19 that was associated with increased complement activation.^15^ Of note, this variant is not mapped in any complement gene coding loci, but it may be indirectly associated with enhanced complement activation during systemic inflammation and organ damage.^42^ Complement variants have been documented by genetic and transcriptional analysis, reporting 23 study‐wide significant single‐nucleotide polymorphisms (SNPs) in 12 complement genes.^16^ An integrative analysis of molecular and functional data revealed four SNPs in three human complement genes (*C3*, *C4BPA*, *C5AR1*) that encode for missense polymorphic variants associated with viral susceptibility.^43^

Considering that these analyses were not able to provide tools for disease severity prediction that could be helpful in clinical practice or a clinical trial setting, our group recently developed an algorithm identifying complement‐related variants in *C3*, *CFH* and *THBD* that predict COVID‐19 severity.^17^ Nevertheless, the prediction rate of this logical algorithm reached values above 80% only in patients not requiring ICU hospitalization and did not incorporate basic features associated with morbidity and mortality, such as age and gender. In the present study, we improved this logical algorithm in order to identify both ICU and non‐ICU patients. Using the updated algorithm, we identified variants in complement‐related genes (*C3*, *CFH*, *CFRH* and *THBD*), among a targeted panel for complement‐related genes, known to be dysregulated in complement‐related disorder. Two of the five critical variants were also the ones that composed the initial algorithm. Based on the five critical variants derived from the updated algorithm, we further implemented an ANN incorporating age and gender. This tool is able to predict not only morbidity but also mortality in COVID‐19 patients.

Indeed, a tool for early and individualized prediction of morbidity and mortality is a largely unmet clinical need not only in this COVID‐19 pandemic but also in several clinical settings. In COVID‐19, ANNs have been only used to predict outbreaks, and cases, deaths and hospital bed occupancy in the short term.^44^, ^45^ However, in the era of precision medicine, individualized genetic data are able to further implement predictions of morbidity and mortality at a very early time point. This prediction has an added value when it concerns early identification of patients that would potentially benefit from a safe and effective therapeutic approach.^46^ In our case of a targeted complement‐related genetic panel, individualized use of complement inhibitors could be of significant benefit in COVID‐19 and other complement‐related disorders. Therefore, the present ANN may serve as a prototype to further expand applications of precision medicine in this field.

The ANN presented in our study also incorporates the effect of age and gender on COVID‐19 morbidity and mortality. Indeed, male COVID‐19 patients show increased morbidity and mortality.^47^ In addition, higher complement activity has been observed in healthy male versus female subjects.^48^ In line with our previous analysis, the present study confirms differences in complement‐related germline variants between male and female COVID‐19 patients. Other factors that may be also associated with differences in morbidity and mortality, such as differences in socioeconomic factors or comorbidities, are also incorporated in the algorithm by the addition of age and gender.

Our study is also the first to show an association between genotype and phenotype in complement dysregulation of COVID‐19 patients. Despite difficulties of functional complement assessment in clinical laboratories,^41^ our results are in line with other functional studies in COVID‐19 showing increased THBD and C3a levels in patients with severe disease.^49^, ^50^ More than that, we also associated impaired levels with the presence of variants recognized as critical in the present study. These data highlight the potential of complement‐related variants to predict complement dysregulation and presumably benefit from complement inhibition. The present study also focusses on alternative pathway variants, since this is directly activated by SARS‐COV‐2.^7^ In terms of complement inhibition, different pathways have been studied. In spite of encouraging results with different inhibitors,^19^, ^20^, ^21^, ^22^ randomized studies have failed to show positive results yet. These could be attributed to several reasons, including study design, patient populations and choice of an appropriate inhibitor based on mechanistic evaluations.^51^ Therefore, broadening complement inhibition in COVID‐19 requires well‐designed studies, as well as appropriate patient selection that would overcome not only methodological but also practical issues of cost and availability during the pandemic. Our study has some limitations. Despite the prospective patient recruitment, our findings cannot be considered as definitive, indicating the need for future studies. In addition, our patient population is rather small, and therefore, the suggested ANN needs to be further validated in other real‐world cohorts.

## 5 CONCLUSIONS

An artificial neural network model was developed, trained and evaluated targeted to the prediction morbidity and mortality ratios of COVID‐19 patients. A number of complement‐related genetic variants, associated with severe COVID‐19, were identified and used as inputs to the model, together with patient's age and gender. Using a sample of 133 patients, the developed ANN model was found capable to successfully predict COVID‐19 severity in 89.47% of the study population.

In conclusion, germline complement‐related genetic variants along with age and gender predict morbidity and mortality in COVID‐19 patients. Given that vaccinations and viral mutations constantly change the landscape of COVID‐19, a prediction tool based on such robust variables is of high importance in the future of this pandemic. Additionally, such a prediction tool is also expected to significantly contribute to better selection of patients that would benefit from targeted complement inhibition, considering the clinical phenotype associated with these variants. Last but not least, this novel approach of artificial intelligence paves the way for future application in additional clinical entities.

## CONFLICTS OF INTEREST

E.G. has consulted for Omeros Cooperation and is supported by the ASH Global Research Award. Remaining authors declare no competing financial interest.

## AUTHOR CONTRIBUTIONS

**Panagiotis Asteris:** Conceptualization (equal); Formal analysis (equal); Software (equal); Supervision (equal); Writing – original draft (equal). **Eleni Gavriilaki:** Conceptualization (equal); Data curation (equal); Funding acquisition (equal); Resources (equal); Writing – original draft (equal). **Tasoula Touloumenidou:** Investigation (equal); Methodology (equal). **Evaggelia‐Evdoxia Koravou:** Methodology (equal). **Maria Koutra:** Methodology (equal). **Penelope Georgia Papayanni:** Methodology (equal). **Alexandros Pouleres:** Investigation (equal). **Vassiliki Karali:** Investigation (equal). **Minas Lemonis:** Software (equal). **Anna Mamou:** Software (equal). **Athanasia Skentou:** Software (equal). **Apostolia Papalexandri:** Resources (equal). **Christos Varelas:** Investigation (equal). **Fani Chatzopoulou:** Methodology (equal). **Maria Chatzidimitriou:** Methodology (equal). **Dimitrios Chatzidimitriou:** Validation (equal). **Anastasia Veleni:** Investigation (equal). **Evdoxia Rapti:** Methodology (equal). **Ioannis Kioumis:** Investigation (equal). **Evaggelos Kaimakamis:** Investigation (equal). **Milly Bitzani:** Investigation (equal). **Dimitrios Boumpas:** Supervision (equal). **Argyris Tsantes:** Supervision (equal). **Damianos Sotiropoulos:** Supervision (equal). **Anastasia Papadopoulou:** Data curation (equal); Methodology (equal). **Ioannis Kalantzis:** Software (equal). **Lydia Vallianatou:** Software (equal). **Danial Armaghani:** Software (equal). **Liborio Cavaleri:** Software (equal). **Amir Gandomi:** Software (equal). **Mohsen Hajihassani:** Software (equal). **Mahdi Hasanipanah:** Resources (equal). **Mohammadreza Koopialipoor:** Resources (equal). **Paulo Lourenco:** Software (equal). **Pijush Samui:** Software (equal). **Jian Zhou:** Software (equal). **Ioanna Sakellari:** Supervision (equal); Writing – review & editing (equal). **Serena I. Valsami:** Methodology (equal); Writing – review & editing (equal). **Marianna Politou:** Project administration (equal); Writing – review & editing (equal). **Styliani Kokoris:** Validation (equal); Visualization (equal); Writing – review & editing (equal). **Achilles Anagnostopoulos:** Validation (equal); Visualization (equal); Writing – review & editing (equal).

## Supporting information

Table S1‐S2

## Data Availability

Data will be immediately available upon communication.
