# Supplementary material for: Genetic prediction of ICU hospitalization and mortality in COVID‐19 patients using artificial neural networks
Source: J Cell Mol Med. 2022 Jan 22;26(5):1445–55. doi: 10.1111/jcmm.17098 (PMC8899198; doi:10.1111/jcmm.17098)
Supplement: Supplementary file 1 — Table S1‐S2 [file JCMM-26-1445-s001.docx]

**Supplementary Tables**

**Supplementary Table 1. Characteristics of the 5 critical variants**

| **Variants** | | | | | **Frequence of Variants** | | |
| --- | --- | --- | --- | --- | --- | --- | --- |
| **rs** | **Gene** | **Position** | **Effect** | **Allele of disease severity** | **Patients not in ICU** | **Patients in ICU** | **p-value** |
| **rs2547438** | C3 | chr1 | Intron | T>G | 28 | 13 | 0.251 |
| **rs2250656** | C3 | chr19 | Intron | T>C | 42 | 20 | 0.112 |
| **rs1042580** | THBD | chr20 | 3 Prime UTR | T>C | 53 | 27 | 0.103 |
| **rs800292** | CFH | chr1 | missense | G>A | 28 | 20 | 0.854 |
| **rs414628** | CFHR1 | chr1 | synonymous | A>T | 38 | 33 | 0.112 |
| THBD: thrombomodulin; CFH: complement factor H; CFHR: complement factor H-related; ICU: intensive care unit; chr1: chromosome 1; UTR: untranslated region | | | | | | | |

**Supplementary Table 2. Training parameters of ANN models**

| **Parameter** | **Value** | **Matlab function** |
| --- | --- | --- |
| Training Algorithm | Levenberg-Marquardt Algorithm | Trainlm |
| Normalization | Minmax in the range 0.10 – 0.90 and -1.00 – 1.00  Zscore | Mapminmax  Zssore |
| Number of Hidden Layers | 1 |  |
| Number of Neurons per Hidden Layer | 1 to 50 by step 1 |  |
| Control random number generation | 10 different random generation | rand(seed, generator), where generator range from 1 to 10 by step 1 |
| Training Goal | 0 |  |
| Epochs | 250 |  |
| Cost Function | Mean Square Error (MSE)  Sum Square Error (SSE) | mse  sse |
| Transfer Functions | Hyperbolic Tangent Sigmoid transfer function (HTS)  Log-sigmoid transfer function (LS)  Linear transfer function (Li)  Positive linear transfer function (PLi)  Symmetric saturating linear transfer function (SSL)  Soft max transfer function (SM)  Competitive transfer function (Co)  Triangular basis transfer function (TB)  Radial basis transfer function (RB)  Normalized radial basis transfer function (NRB) | tansig  logsig  purelin  poslin  satlins  softmax  compet  tribas  radbas  radbasn |
